# Association analyses of the interaction between the *ADSS *and *ATM *genes with schizophrenia in a Chinese population

**DOI:** 10.1186/1471-2350-9-119

**Published:** 2008-12-30

**Authors:** Fuquan Zhang, Yong Xu, Pozi Liu, Hua Fan, Xuezhu Huang, Gaoxiang Sun, Yuqing Song, Pak C Sham

**Affiliations:** 1 Institute of Neurological disorders, Tsinghua University, Department of Psychiatry, Yuquan Hospital, Tsinghua University, Bejing, 100049, PR China; 2 Department of Psychiatry, Beijing Anding Hospital, Capital Medical University, Bejing, PR China; 3 Department of Psychiatry, University of Hong Kong, Hong Kong, PR China

## Abstract

**Background:**

The blood-derived RNA levels of the adenylosuccinate synthase (*ADSS*) and ataxia telangiectasia mutated (*ATM*) genes were found to be down- and up-regulated, respectively, in schizophrenics compared with controls, and *ADSS *and *ATM *were among eight biomarker genes to discriminate schizophrenics from normal controls. ADSS catalyzes the first committed step of AMP synthesis, while ATM kinase serves as a key signal transducer in the DNA double-strand breaks response pathway. It remains unclear whether these changes result from mutations or polymorphisms in the two genes.

**Methods:**

Six SNPs in the *ADSS *gene and three SNPs in the *ATM *gene in a Chinese population of 488 schizophrenics and 516 controls were genotyped to examine their association with schizophrenia (SZ). Genotyping was performed using the Sequenom platform.

**Results:**

There was no significant difference in the genotype, allele, or haplotype distributions of the nine SNPs between cases and controls. Using the Multifactor Dimensionality Reduction (MDR) method, we found that the interactions among rs3102460 in the *ADSS *gene and rs227061 and rs664143 in the *ATM *gene revealed a significant association with SZ. This model held a maximum testing accuracy of 60.4% and a maximum cross-validation consistency of 10 out of 10.

**Conclusion:**

These findings suggest that the combined effects of the polymorphisms in the *ADSS *and *ATM *genes may confer susceptibility to the development of SZ in a Chinese population.

## Background

Schizophrenia (SZ) is a complex genetic disorder characterized by profound disturbances of cognition, emotion, and social functioning. Numerous family, twin, and adoption studies conclusively show that SZ risk is increased among the relatives of affected individuals and that SZ is largely the result of genes rather than shared environment [1], the estimated heritability of SZ is 80–85% [2]. Besides traditional association or linkage studies, recent advances have facilitated the use of circulating blood to conduct genomic analyses of human diseases [3,4]; the search for the genetic basis of SZ has produced some lines of evidence at the level of gene expression. By analyzing the blood-derived RNA from 74 samples, linear and nonlinear combinations of eight putative biomarker genes (*APOBEC3B, ADSS, ATM, CLC, CTBP1, DATF1, CXCL1*, and *S100A9*) were able to discriminate between SZ, bipolar disorder, and control samples [5], with an overall accuracy of 95 – 97%. As yet, none of these genes have been investigated for their association or linkage disequilibrium (LD) with SZ.

It has been shown that adenylosuccinate synthase (*ADSS*) is downregulated, while ataxia telangiectasia mutated (*ATM*) is upregulated, in schizophrenics [5]. The de novo biosynthesis of AMP from IMP involves two steps: the first step is catalyzed by ADSS, and the second step is catalyzed by adenylosuccinate lyase [6]. *ADSS *can influence energy metabolism through the purine nucleotide cycle and the AMP-activated protein kinase (AMPK) pathway [7]. Cyclic AMP (cAMP), a very close structural relative of AMP, containing an additional ester linkage between the phosphate and ribose units, acts as a secondary messenger for several hormones. It is tempting to postulate that *ADSS *may play a role in the pathogenesis of the illness via energy metabolism or nucleotide synthesis.

DNA damage poses a continuous threat to genomic integrity in mammalian cells, with the most deleterious form being double-strand breaks (DSBs). ATM signaling is required to sense and initiate repair of DSBs. When DSBs occur, ATM initiates a well-characterized response to DNA damage, resulting in cell-cycle arrest, DNA repair, or apoptosis. In this way, ATM functions as a fundamental safeguard against genomic instability during organism development. ATM and ATR (ATM and Rad3-related) substrate analysis revealed extensive protein networks responsive to DNA damage, involving more than 900 regulated phosphorylation sites encompassing over 700 proteins [8]. Thus ATM may be a possible candidate gene underlying SZ.

cAMP response element binding protein is phosphorylated by ATM on Ser-121 in response to ionizing radiation and oxidative stress [9]. ATM has been shown to phosphorylate the AMPK α subunit [10], and ATM-dependent mitochondrial biogenesis is mediated through AMPK [11]. These studies provide evidence of their interaction in some pathways possibly affecting brain function. Therefore, we hypothesized that there may be interactions between these two genes conferring disease risk for SZ, but it is unknown whether genetic variation underlies the alterations in *ADSS *and *ATM *expression. To test the hypothesis that sequence variations in the *ADSS *or *ATM *genes influence risk for the disease, we conducted a case-control association study on nine SNPs within the two genes in a Chinese Han population. Frequency of alleles, genotypes, and haplotypes of the nine SNPs were tested between cases and controls.

Because SZ is a common disease with a complex multifactorial etiology, several recent approaches are promising for detecting gene-gene and gene-environment interactions. Multifactor Dimensionality Reduction (MDR) is a data reduction method for detecting multilocus genotype combinations that predict disease risk for common, complex disease [12-14]. MDR pools genotypes into "high risk" and "low risk" groups to reduce multidimensional data into only one dimension. A certain threshold, defined as the ratio of cases to controls, determines the risk group to which a factor combination is assigned [15]. Using MDR, many studies have observed that complex interactions among multiple genes may make a genetic contribution to complex disorders [16-18], including SZ [19-21]. Here, we explored the epistasis, or gene × gene interaction, between the two genes via MDR.

## Methods

### Subjects

A total sample of 488 unrelated SZ patients and 516 healthy controls was collected. Cases were recruited from Hong Kong hospitals. All patients were interviewed using the Structured Clinical Interview for DSM-IV and met the DSM-IV diagnostic criteria for SZ.

Healthy controls were recruited from blood donors who were not screened for psychiatric diseases; however, in Hong Kong an individual would be ineligible for blood donation if he is under a doctor's care, taking medications, awaiting test results, or suffering from any serious illness. All subjects were Han Chinese. Peripheral blood sample were obtained from the subjects. The present study was approved by the Institutional Review Board of the University of Hong Kong/Hospital Authority Hong Kong West Cluster, and written informed consent was obtained from all subjects.

### Genotyping

Based on the location and the heterozygosity of the SNPs, we selected six SNPs (rs3102460, rs3127459, rs3127460, rs3127465, rs3006001, and rs3003211) in the *ADSS *gene and three SNPs (rs600931, rs227061, and rs664143) in the *ATM *gene to check their allelic and haplotypic association to SZ in a case-control sample.

We used a Sequenom platform (Sequenom MassARRAY System, Sequenom, San Diego, CA, USA) for assay design and genotyping. SNP sites were amplified by PCR in multiplex format in 384-well microtiter plates by a pair of specifically designed forward and reverse PCR primers. The length of the amplicons for the SNP capture ranged from 60 to 120 base pairs. Following genomic amplification of the target regions, PCR products were treated with shrimp alkaline phosphatase for 20 min at 37°C to dephosphorylate any residual nucleotides and to prevent their future incorporation and interference with the primer extension assay. Extension primers, DNA polymerase, and a cocktail mixture of deoxynucleotides and dideoxynucleotide triphosphates were added to each mix. These were then followed by cycles of homogeneous MassEXTEND reaction probed by the extension primers for each SNP. The MassARRAY typer software (version 3.1) was then used to read out the extended mass and assign the genotype call. Quality control criteria included a genotype call rate of > 80%, less than 1 duplicate error (5 duplicates in each 96-well plate), and significant Hardy-Weinberg disequilibrium.

### Statistical analyses

Hardy-Weinberg equilibrium and genotype and allele frequencies between cases and controls were tested using PLINK-1.05 [22]. LD between markers was tested with Haploview version 4.1 (Barrett, 2005). Haplotype analyses were performed using UNPHASED (version 3.0.5) [23], as well as SHEsis [24]. Haplotypes with frequencies < 3% in the whole sample were considered to be rare and were excluded. The gene-gene interactions were analyzed by MDR. Ten-fold cross-validation was used in our MDR analysis. Data were randomly split into 10 approximately equal parts; one subdivision was used as the testing set and the rest as the training set. In view of our data, we considered two- to four-locus interaction models, performing 1,000 permutations.

## Results

### Genotype and allele distributions of SNPs

The distributions of allele and genotype frequencies of nine SNPs among 488 SZ patients and 516 healthy controls are shown in Table 1. The genotypic distributions of these nine polymorphisms do not deviate significantly from Hardy-Weinberg equilibrium in both patients and controls (data not shown). There was no significant difference in genotype or allele frequencies between cases and controls.

**Table 1:** Genotypic and allelic distributions of the 9 SNPs in cases and controls

SNP		Genotype Frequency (%) p(df = 2)			Allele Frequency (%) p(df = 1) OR(95%CI)	
		CC	CT	TT	C	T
SNP1:rs3102460	ca 441	48(10.9) 176(39.9) 217(49.2) 0.435			272(30.8) 610(69.2) 0.329 1.10(0.90–1.35)	
	co 444	37(8.3) 181(40.8) 226(50.9)			255(28.7) 633(71.3)	
		AA	AT	TT	A	T
SNP2:rs3127459	ca 481	26(5.4) 172(35.8) 283(58.8) 0.404			224(23.3) 738(76.7) 0.214 0.88(0.71–1.08)	
	co 508	37(7.3) 187(36.8) 284(55.9)			261(25.7) 755(74.3)	
		TT	AT	AA	T	A
SNP3:rs3127460	ca 482	12(2.5) 151(31.3) 319(66.2) 0.720			175(18.2) 789(81.8) 0.463 0.92(0.74–1.16)	
	co 504	16(3.2) 164(32.5) 324(64.3)			196(19.4) 812(80.6)	
		CC	CT	TT	C	T
SNP4: rs3127465	ca 474	11(2.3) 144(30.4) 319(67.3) 0.697			166(17.5) 782(82.5) 0.595 0.93(0.74–1.18)	
	co 499	16(3.2) 152(30.5) 331(66.3)			184(18.4) 814(81.6)	
		CC	AC	AA	C	A
SNP5: rs3006001	ca 481	12(2.5) 148(30.8) 321(66.7) 0.636			172(17.9) 790(82.1) 0.362 0.91(0.72–1.14)	
	co 503	16(3.2) 164(32.6) 323(64.2)			196(19.5) 810(80.5)	
		GG	AG	AA	G	A
SNP6: rs3003211	ca 436	50(11.5) 171(39.2) 215(49.3) 0.724			271(31.1) 601(68.9) 0.455 1.09(0.89–1.34)	
	co 455	45(9.9) 178(39.1) 232(51)			268(29.5) 642(70.5)	
		AA	AG	GG	A	G
SNP7: rs600931	ca 479	82(17.1) 231(48.2) 166(34.7) 0.556			395(41.2) 563(58.8) 0.462 1.06(0.89–1.28)	
	co 500	73(14.6) 250(50) 177(35.4)			396(39.6) 604(60.4)	
		GG	AG	AA	G	A
SNP8: rs227061	ca 481	82(17.0) 231(48) 168(34.9) 0.496			395(41.1) 567(58.9) 0.465 0.94(0.78–1.13)	
	co 502	72(14.3) 252(50.2) 178(35.5)			396(39.4) 608(60.6)	
		CC	CT	TT	C	T
SNP9: rs664143	ca 483	92(19.0) 227(47) 164(34) 0.163			411(42.5) 555(57.5) 0.950 1.01(0.84–1.20)	
	co 507	81(16.0) 268(52.9) 158(31.2)			430(42.4) 584(57.6)	

ca = case; co = control.

### Patterns of LD

The patterns of pairwise LD between neighboring SNPs are shown in Table 2. D primes range from approximately 0.96 to 1. Fairly tight LD was observed in any pair of the 1–6 SNPs in *ADSS *and the 7–9 SNPs in *ATM*, and SNPs of each gene were in strong LD.

**Table 2:** Pairwise linkage disequilibrium (LD) of the SNPs within each gene

D'	SNP2	SNP3	SNP4	SNP5	SNP6	SNP8	SNP9
SNP1	0.99	0.96	1	0.98	0.99		
SNP2		0.98	0.99	0.99	0.99		
SNP3			0.99	0.99	0.96		
SNP4				1	1		
SNP5					0.98		
SNP7						0.99	1
SNP8							0.99

### Haplotypes of the SNPs in ADSS and ATM

There was no individual or global significant difference for the 6-marker haplotypes in *ADSS *or the 3-marker haplotypes in *ATM *between cases and controls (Table 3). Individual haplotype tests were performed by evaluating the risk difference between a specific haplotype and all others grouped together.

**Table 3:** Estimated haplotype frequencies and association significance of *ADSS *and *ATM*

Gene	SNP	Haplotype	Case (freq%) Control (freq%)	X^2 ^p	OR (95%CI)	Global p
ADSS	SNP1-SNP6	TTATAA	372.0(46.2) 364.6(45)	0.119 0.731	1.04 (0.85–1.26)	0.339
		CTATAG	243.0(30.1) 221.4(27.3)	1.388 0.239	1.14 (0.92–1.41)	
		TATCCA	140.0(17.4) 156.0(19.3)	1.087 0.297	0.87 (0.68–1.13)	
		TAATAA	45.0(5.6) 57.0(7.0)	1.523 0.217	0.78 (0.52–1.16)	
ATM	SNP7-SNP9	A G C	384.0(40.8) 377.0(39.3)	0.254 0.613	1.05 (0.87–1.26)	0.600
		G A T	538.0(57.1) 554.0(57.7)	0.254 0.615	0.95 (0.79–1.15)	

Freq = frequency. Haplotypes were omitted from analysis if the estimated haplotype probabilities were less than 3%

### Gene-gene interactions among ADSS and ATM

**Figure 1:**
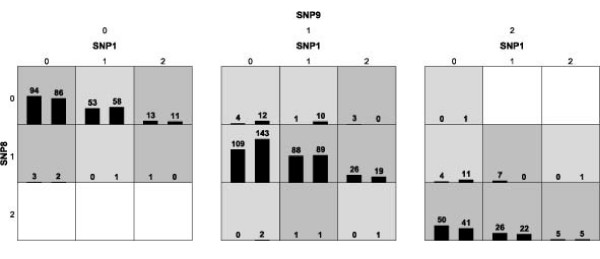
**The best three-locus SNP model selected by MDR**. 0, homozygote of common allele; 1, heterozygote; 2, homozygote of rare allele. High risk combinations are depicted as dark-shaded cells; low-risk combinations are depicted as light-shaded cells; empty cells are left blank. For each cell, the left bar indicates the total number of cases, and the right bar indicates the total number of controls.

Gene-gene interactions of the nine SNPs were examined using the MDR method, and the results for each number of factors considered are summarized in Table 4. We tested 2- to 4-locus combinations within the two genes. Because the MDR procedure works best with the model that has the maximum cross-validation consistency and minimum prediction error, the three locus model, involving rs3102460 in the *ADSS *gene as well as rs227061 and rs664143 in the *ATM *gene, had a cross-validation consistency of 10, being regarded as the best (*p *= 0.011). The 1000-fold permutations test showed that *p *was larger than 0.05 (Figure 1).

**Table 4:** The interaction models detected by MDR

model	training bal. acc.	testing bal. acc.	Sign test (p)	CV consistency
SNP 9	0.5307	0.5307	7 (0.172)	10/10
SNP 1, 9	0.5476	0.4989	3 (0.945)	7/10
SNP 1, 8, 9	0.5593	0.5260	9 (0.011)*	10/10
SNP 1, 7, 8, 9	0.5638	0.5199	8 (0.055)	10/10

bal. acc.: Balanced Accuracy. *: p > 0.05 based on 1000 permutations

## Discussion

Besides several very promising candidate genes for SZ, such as *NRG1 *[25], *DTNBP1 *[26], *COMT *[27], *DISC1 *[28], and *DAOA *[29,30], there may also be genes that play subtle or weak roles in the pathogenesis of SZ, making them difficult to identify by traditional approaches. An alternative approach is the use of microarray technology to examine differential RNA gene expression between patients and controls, by which *ADSS *and *ATM *were suggested as biomarker genes for SZ [5]. Although the mechanism underlying the alterations is unknown, the results suggest that the *ADSS *and *ATM *genes may be involved in the genetic architecture of SZ, since genetic polymorphisms within the genes may influence gene expression.

The gene encoding *ADSS *maps to 1cen-q12, a chromosomal locus previously linked to SZ by meta-analysis [31,32]. Similarly, *ATM*'s genomic location, 11q22–23, was reported to be one of the genetic susceptibility regions by meta-analysis [1,32-36] and several other independent studies [33-36]. This region also contains another controversial risk gene, *DRD2 *(dopamine receptor D2) [37,38].

The *ADSS *gene is 44 kb in length with 13 exons. Six SNPs from intron 11 (rs3102460), intron 6 (rs3127459), intron 4 (rs3127460), and intron 1 (rs3127465, rs3006001, and rs3003211) in the *ADSS *gene were selected for the current study. Among them, rs3102460, rs3127465, and rs3006001 were predicted to influence transcriptional regulation; no functional information exists for the remaining three SNPs yet [39]. The *ATM *gene is 146 kb in length, consisting of 63 exons. The three SNPs in *ATM *are from intron 7 (rs600931), intron 54 (rs227061), and intron 61 (rs664143); rs227061 and rs664143 possibly affect transcriptional regulation [39]. Our data did not yield any statistically significant difference for genotype, allele, or haplotype distributions between cases and controls.

Epistasis, or gene × gene interaction, is increasingly assumed to play a crucial role in the genotype-to-phenotype relationship of common diseases [40]. Although the ubiquity of joint actions appears to be a natural property of complex traits, the nature of joint actions has not been well investigated or understood. To our knowledge, this is the first genetic study to test the joint action of the *ADSS *and *ATM *genes in relation to SZ. The interactions between *ADSS *and *ATM *were assessed using the MDR program, which has been widely used for detecting epistasis in complex human diseases. The combined effects of the polymorphisms in *GRIN1 *and *GRIN2B *[19], as well as the combined effects of *GAD1*, *GAD2*, and *GABRB2 *[21], were found to be associated with SZ in a Chinese population. Yasuno etc. [20] suggested that synergistic interaction between *UCP *(uncoupling protein) 2 and *UCP4 *may be involved in the etiology of SZ in a Japanese population. In our analysis, the three-locus model (rs3102460 in the *ADSS *gene and rs227061 and rs664143 in the *ATM *gene) was selected as the best one for determining SZ susceptibility based on its balanced accuracy and cross-validation consistency, which suggests that the interactions among these SNPs may be associated with SZ. An interaction dendrogram from the MDR demonstrated a strong synergic interaction between SNP1 and SNP8, suggesting a combined effect between the two genes; however, the result was not robust enough to survive correction of permutation test, indicating the need for larger samples to validate our result. Nevertheless, based on the cross-validation consistency and testing accuracy, the results could partially support the hypothesis that some loci contribute to a certain complex disease only through interaction with other genes (epistasis), while the main effects of the individual locus may be small or absent [41]. Detection of an interaction between the two genes is potentially novel and intriguing from a biological perspective because it suggests the attractive implication that an impediment of DNA repair may play a role in the abnormal neurodevelopment in SZ. Such predictions deserve to be validated experimentally using systems biology approaches and animal models.

This study sought to explore the genetic basis of SZ using clues from RNA alteration. Although it did not support *ADSS *or *ATM *as an individual candidate gene for the illness, the study suggested that the epistatic effect of a three-locus interaction within the *ADSS *and *ATM *genes may exist for SZ susceptibility. One common issue in the study of complex diseases is the limited sample size, resulting in inadequate power to detect association. Assuming the frequency of risk allele in controls to be 0.5, our sample of 488 cases and 516 controls is able to detect an odds ratio of 1.37 or above with 80% power [42]. With regard to our data, the absence of main effects of polymorphisms in *ADSS *or *ATM *may arise from insufficient power. Similarly, Qin etc. [19] detected interactions between the *GRIN1 *and *GRIN2B *genes in the absence of main effects of a single marker in SZ.

Case-control studies are susceptible to positive and negative artifacts from unknown population stratifications or different levels of ethnic admixture among cases and controls. Family-based association studies are helpful to circumvent stratification biases, so it is necessary to examine the transmission from parents to affected offspring in future studies. Moreover, because different populations have distinct genetic backgrounds, it is necessary to validate or replicate our association results using independent samples, especially from other ethnic populations. Our data should be interpreted with caution, considering it was a statistical epistasis. Therefore, experiments demonstrating the mechanisms by which alterations in these two genes in tandem can cause brain and behavioral changes associated with SZ would provide the most vital support for our hypothesis.

## Conclusion

In spite of potential limitations, the results of our study show that the combined actions of the polymorphisms in the *ADSS *and *ATM *genes may confer a risk for the development of SZ in a Chinese population. Larger sample studies involving more SNPs within the two genes, as well as neurobiological experiments implicating their role in SZ, are needed to validate our results.

## Abbreviations

APOBEC3B: apolipoprotein B mRNA editing enzyme, catalytic polypeptide-like 3B; CLC: Charcot-Leyden crystal protein; COMT: catechol-O-methyltransferase; CTBP1: C-terminal binding protein 1; CXCL1: chemokine (C-X-C motif) ligand 1; DAOA: D-amino acid oxidase activator; DIDO1: death inducer-obliterator 1; DISC1: disrupted in schizophrenia 1; DTNBP1: dystrobrevin binding protein 1; NRG1: neuregulin 1; S100A9: S100 calcium binding protein A9.

## Competing interests

The authors declare that they have no competing interests.

## Authors' contributions

SP designed and supervised the study. ZF, XY and LP drafted the manuscript. FH, SY, SG and HX participate in data analysis. All authors read and approved the final manuscript.

## Pre-publication history

The pre-publication history for this paper can be accessed here:


